# Supplementary figures and images for: In a model of parasite-mediated exhaustion, stem-like CD8 T cells differentiate into an unconventional intermediate effector memory subset
Source: bioRxiv. 2025 Jan 28:2024.10.30.621158. Preprint. [Version 2] doi: 10.1101/2024.10.30.621158 (PMC11838209; doi:10.1101/2024.10.30.621158)

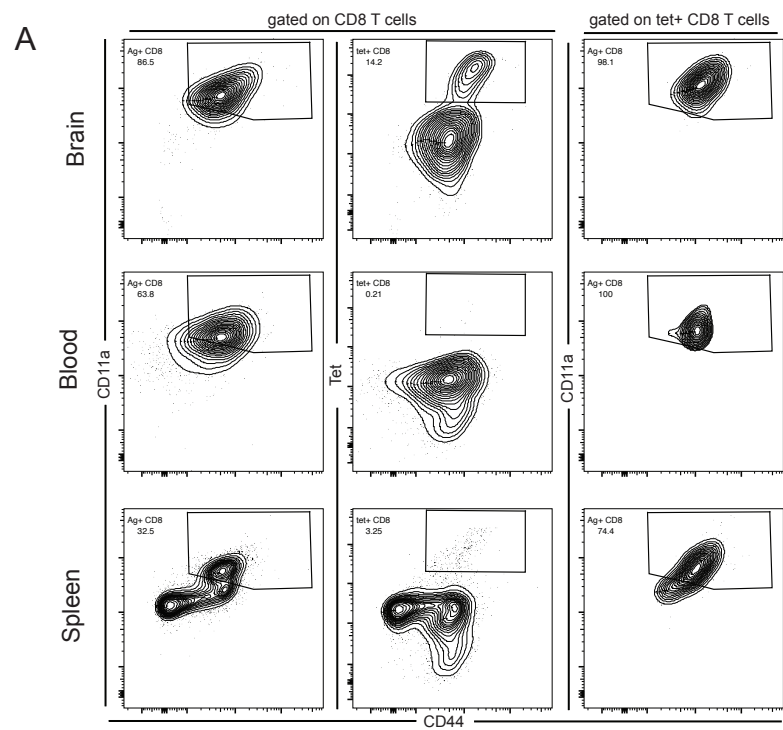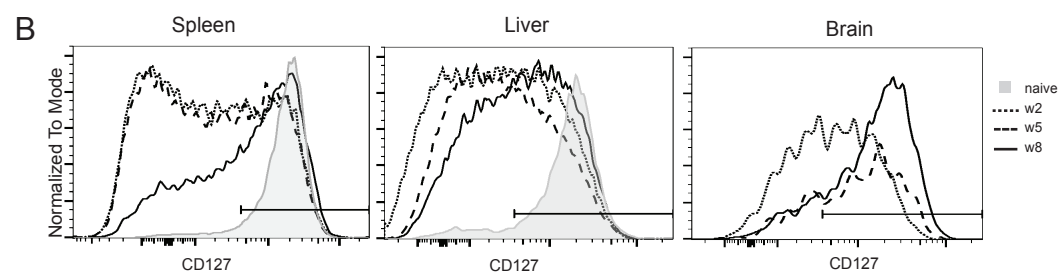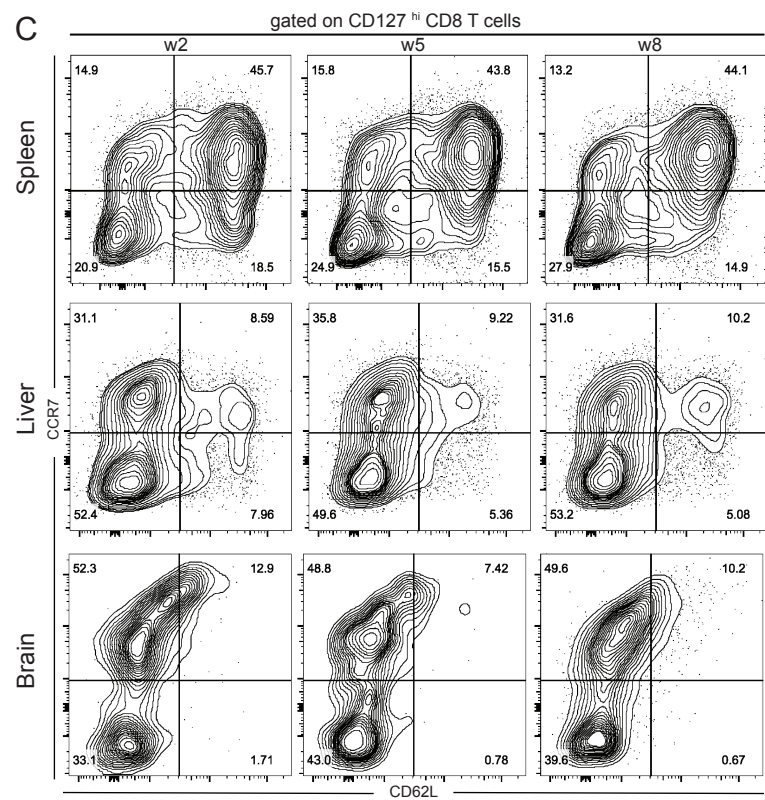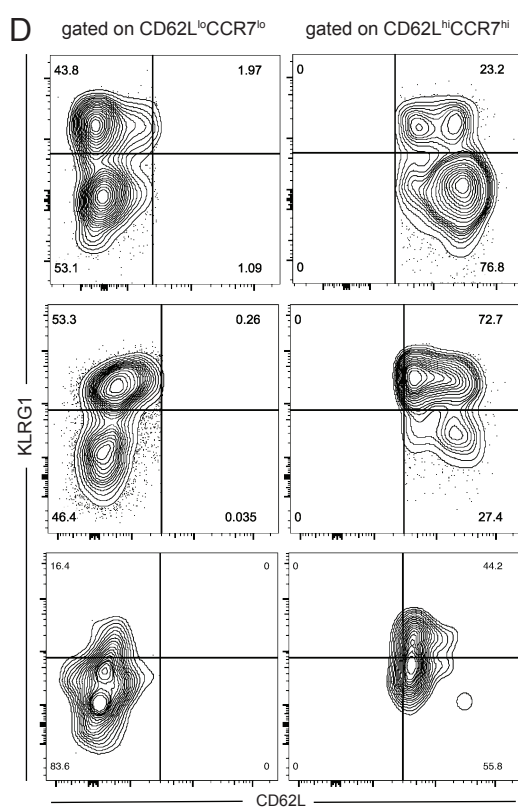

Supplement: Supplement 1 [file media-1.pdf]

A

w2 p.i.

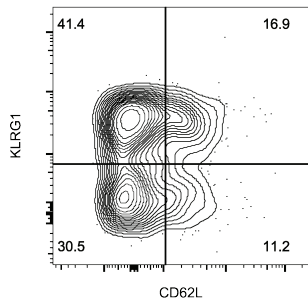

w2 p.i.

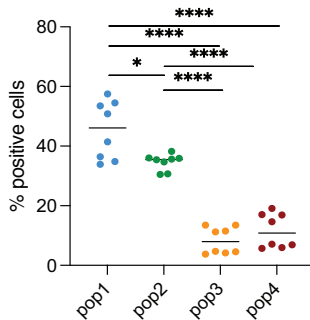

w8 p.i.

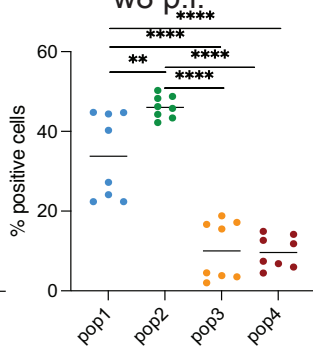

B

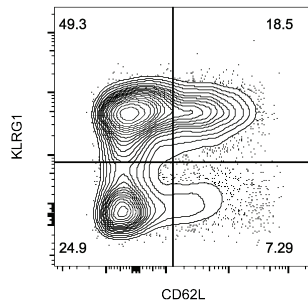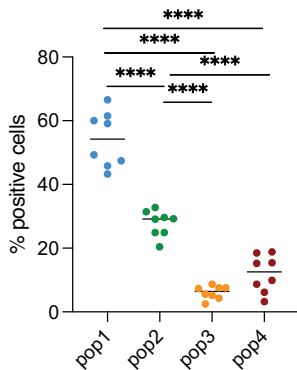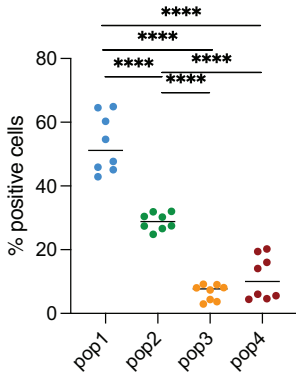

Supplement: Supplement 2 [file media-2.pdf]

# Brain

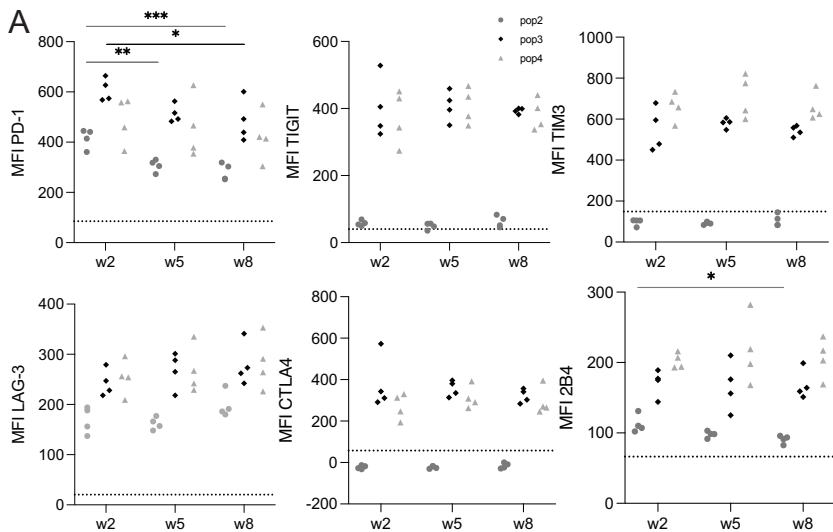

# Liver

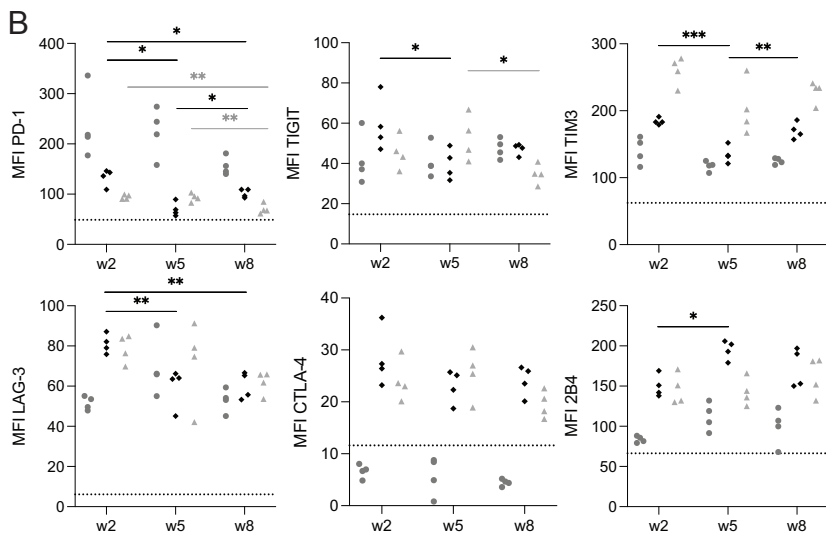

Supplement: Supplement 3 [file media-3.pdf]

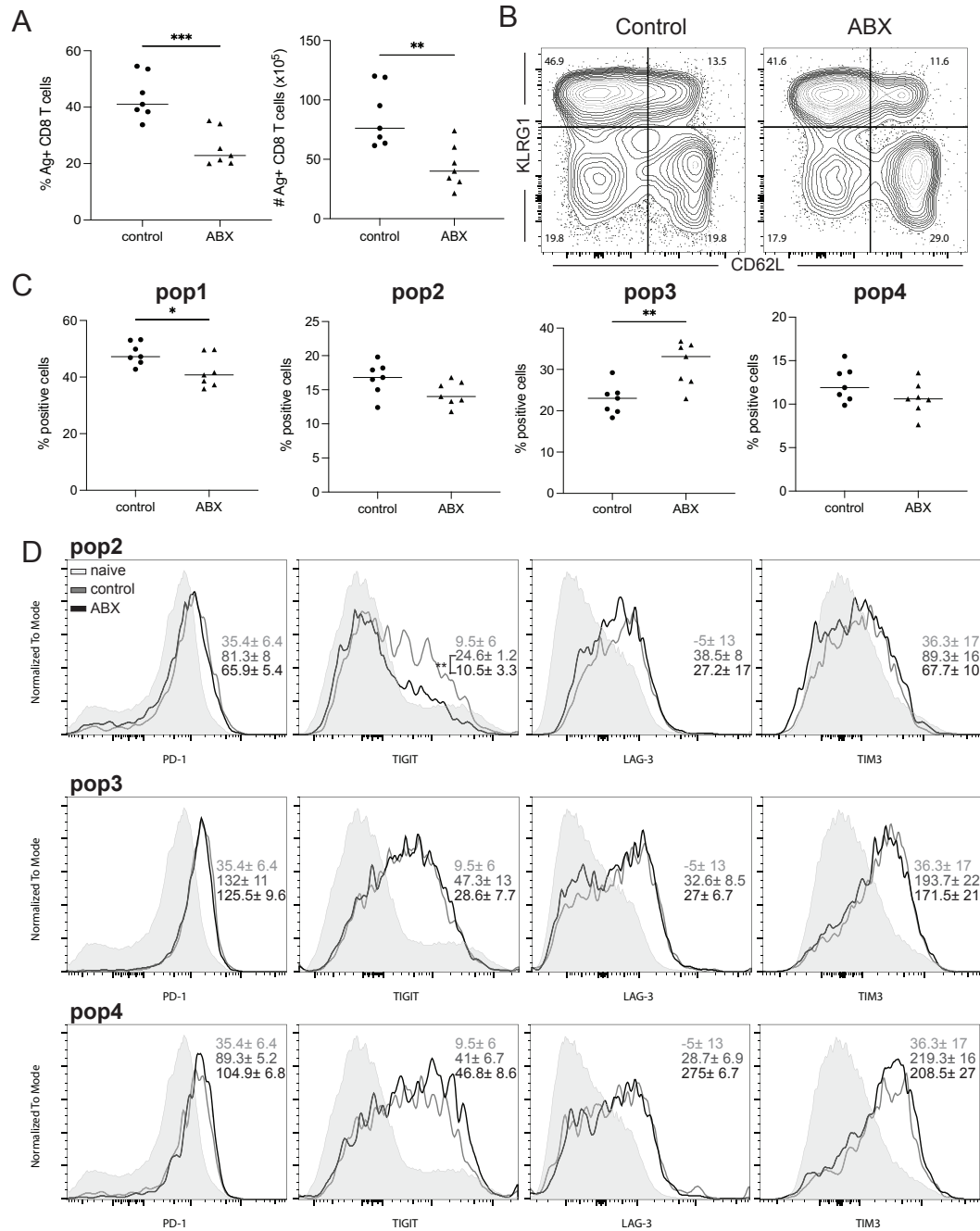

Supplement: Supplement 4 [file media-4.pdf]

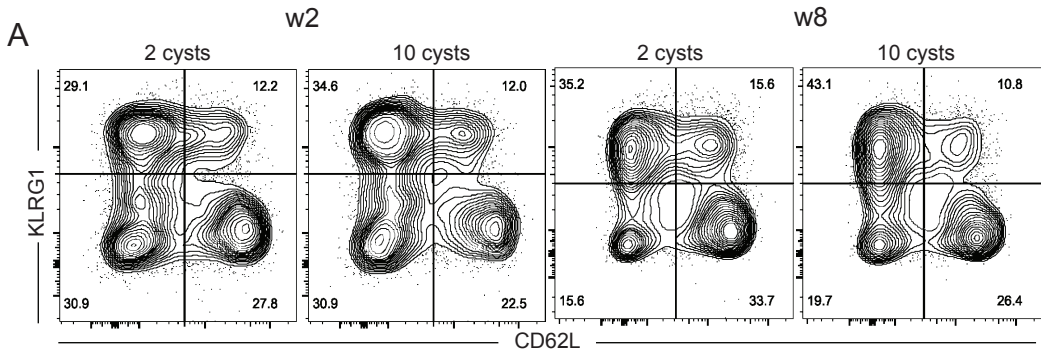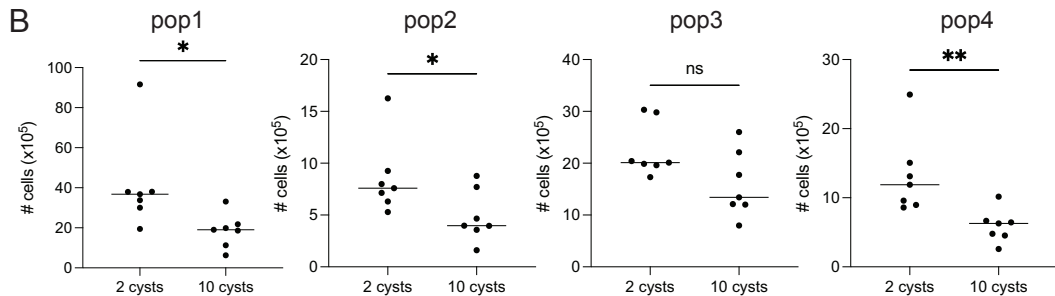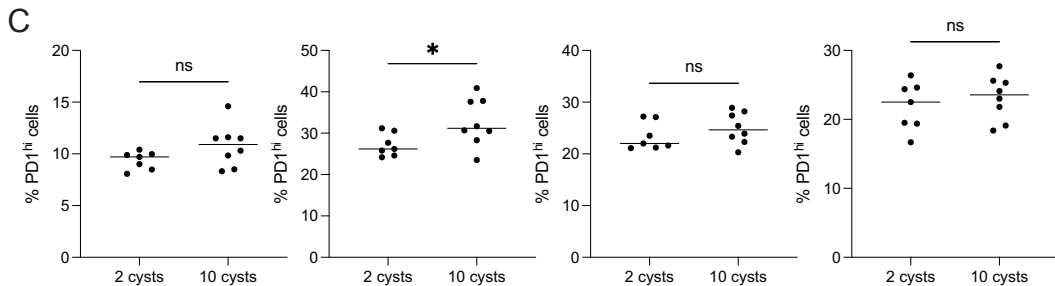

Supplement: Supplement 5 [file media-5.pdf]

**A**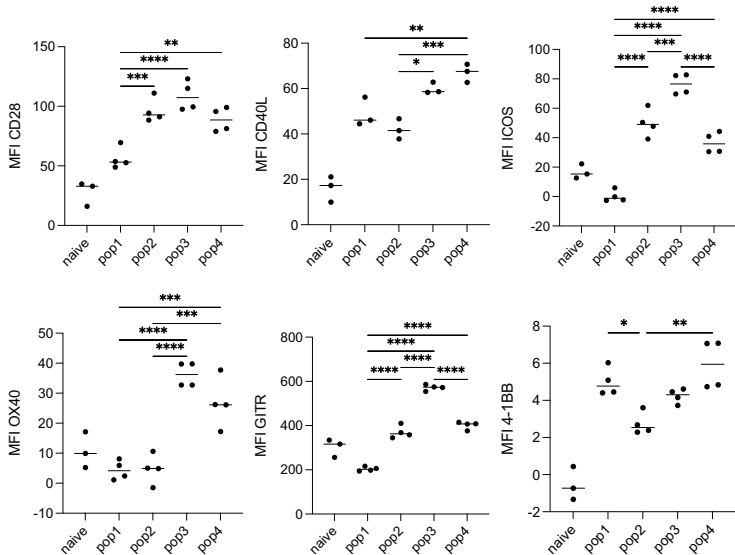**B**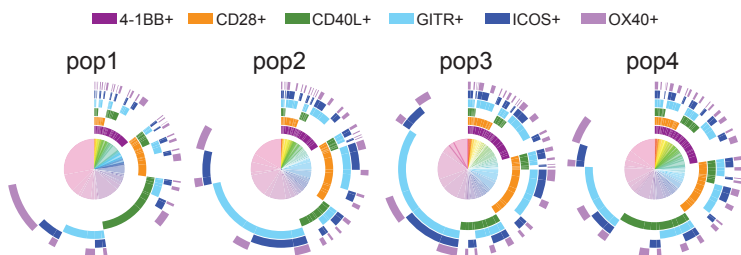**C**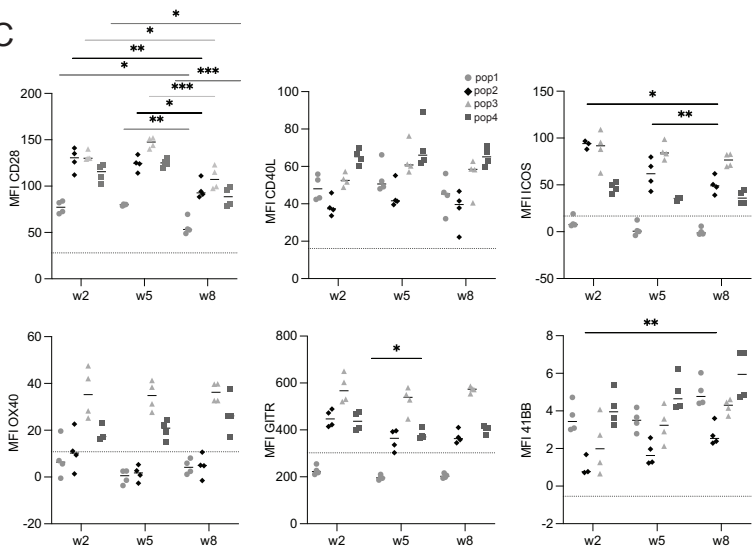

Supplement: Supplement 6 [file media-6.pdf]
