# Supplementary material for: In a model of parasite-mediated exhaustion, stem-like CD8 T cells differentiate into an unconventional intermediate effector memory subset
Source: bioRxiv. 2025 Jan 28:2024.10.30.621158. Preprint. [Version 2] doi: 10.1101/2024.10.30.621158 (PMC11838209; doi:10.1101/2024.10.30.621158)
Supplement: Supplementary file 2 [file media-7.docx]

Cluster	Symbol
1	Mgrn1
1	Degs2
1	Serpina3f
1	Klhl3
1	Lag3
1	Serpina3g
1	Jdp2
1	Cntnap1
1	Egr2
1	Slc45a4
1	Shisa5
1	Snx29
1	Tmem9
1	Sptbn2
1	Cd7
1	Itgae
1	Chrne
1	Plscr3
1	Mfsd2b
1	Ighg2c
1	A3galt2
1	Fam129c
1	Nlrp1b
1	6720464F
1	Myh7b
1	Asb2
1	Jun
1	Axl
1	Cd101
1	Synpo
1	Zmiz2
1	Bcr
1	Ndnf
1	Adamts7
1	Tmem154
1	Bcl6
1	Myo1e
1	Arhgap39
1	Serpina9
1	Inpp5a
[illegible]	[illegible]
