# Supplementary material for: In a model of parasite-mediated exhaustion, stem-like CD8 T cells differentiate into an unconventional intermediate effector memory subset
Source: bioRxiv. 2025 Jan 28:2024.10.30.621158. Preprint. [Version 2] doi: 10.1101/2024.10.30.621158 (PMC11838209; doi:10.1101/2024.10.30.621158)
Supplement: Supplementary file 3 [file media-8.docx]

SYMBOL	GENENAME	ENTREZID	Ensembl ID
Tcf7	transcription factor 7, T cell specific	21414	ENSMUSG00000000782
Id3	inhibitor of DNA binding 3	15903	ENSMUSG00000007872
Myb	myeloblastosis oncogene	17863	ENSMUSG00000019982
Eomes	eomesodermin	13813	ENSMUSG00000032446
Bach2	BTB and CNC homology, basic leucine zipper trans	12014	ENSMUSG00000040270
Tox	thymocyte selection-associated high mobility group	252737	ENSMUSG00000041272
Il7r	interleukin 7 receptor	16197	ENSMUSG00000003882
Sell	selectin, lymphocyte	20343	ENSMUSG00000026581
Ccr7	C-C motif chemokine receptor 7	12774	ENSMUSG00000037944
Pdcd1	programmed cell death 1	18566	ENSMUSG00000026285
Lag3	lymphocyte-activation gene 3	16768	ENSMUSG00000030124
Cd200	CD200 molecule	17470	ENSMUSG00000022661
Cxcr5	C-X-C motif chemokine receptor 5	12145	ENSMUSG00000047880
Slamf6	SLAM family member 6	30925	ENSMUSG00000015314
Nt5e	5' nucleotidase, ecto	23959	ENSMUSG00000032420
Cd69	CD69 antigen	12515	ENSMUSG00000030156
Lef1	lymphoid enhancer binding factor 1	16842	ENSMUSG00000027985
Zeb1	zinc finger E-box binding homeobox 1	21417	ENSMUSG00000024238
Bcl2	B cell leukemia/lymphoma 2	12043	ENSMUSG00000057329
Egr2	early growth response 2	13654	ENSMUSG00000037868
Cd27	CD27 antigen	21940	ENSMUSG00000030336
Cxcr3	C-X-C motif chemokine receptor 3	12766	ENSMUSG00000050232
Tlr1	toll-like receptor 1	21897	ENSMUSG00000044827
